# The Gene Expression Profile of CD11c^+^CD8α^−^ Dendritic Cells in the Pre-Diabetic Pancreas of the NOD Mouse

**DOI:** 10.1371/journal.pone.0103404

**Published:** 2014-08-28

**Authors:** Wouter Beumer, Jojanneke M. C. Welzen-Coppens, Cornelia G. van Helden-Meeuwsen, Sinead M. Gibney, Hemmo A. Drexhage, Marjan A. Versnel

**Affiliations:** Department of Immunology, Erasmus MC, University Medical Center, Rotterdam, The Netherlands; University of Bergen, Norway

## Abstract

Two major dendritic cell (DC) subsets have been described in the pancreas of mice: The CD11c^+^CD8α^−^ DCs (strong CD4+ T cell proliferation inducers) and the CD8α^+^CD103^+^ DCs (T cell apoptosis inducers). Here we analyzed the larger subset of CD11c^+^CD8α^−^ DCs isolated from the pancreas of pre-diabetic NOD mice for genome-wide gene expression (validated by Q-PCR) to elucidate abnormalities in underlying gene expression networks. CD11c^+^CD8α^−^ DCs were isolated from 5 week old NOD and control C57BL/6 pancreas. The steady state pancreatic NOD CD11c^+^CD8α^−^ DCs showed a reduced expression of several gene networks important for the prime functions of these cells, i.e. for cell renewal, immune tolerance induction, migration and for the provision of growth factors including those for beta cell regeneration. A functional *in vivo* BrdU incorporation test showed the reduced proliferation of steady state pancreatic DC. The reduced expression of tolerance induction genes (CD200R, CCR5 and CD24) was supported on the protein level by flow cytometry. Also previously published functional tests on maturation, immune stimulation and migration confirm the molecular deficits of NOD steady state DC. Despite these deficiencies NOD pancreas CD11c^+^CD8α^−^ DCs showed a hyperreactivity to LPS, which resulted in an enhanced pro-inflammatory state characterized by a gene profile of an enhanced expression of a number of classical inflammatory cytokines. The enhanced up-regulation of inflammatory genes was supported by the *in vitro* cytokine production profile of the DCs. In conclusion, our data show that NOD pancreatic CD11c^+^CD8α^−^ DCs show various deficiencies in steady state, while hyperreactive when encountering a danger signal such as LPS.

## Introduction

Diabetes mellitus type 1 (T1DM) is caused by an autoimmune reaction to the islets of Langerhans in the pancreas resulting in an autoimmune insulitis, in which the beta cells disappear with as consequence an absolute insulin deficiency.

The NOD mouse model is considered an excellent model of human T1DM and spontaneously develops an autoimmune insulitis similar to T1DM patients [1]–[3]. With regard to the early phases of the NOD autoimmune insulitis Diana *et al*. recently showed a transient accumulation of a small number of plasmacytoid DCs, lymphocytes and B-cells in the pancreatic islets of NOD mice at 2 weeks of age [4]. An interaction between these infiltrating cells was shown to be involved in the onset of autoimmunity against the beta cells. For this very early time point of 2 weeks of age small transient accumulations of conventional DCs (cDCs) and macrophages around the islets have been reported on before [5], [6], as well as on apoptosis abnormalities in the pancreas of the NOD mouse [7].

This first relatively mild intra-islet and peri-islet accumulation of immune cells at 2 weeks of age is followed by a second wave of a larger para- and peri-islet immune cell accumulation starting at 5 weeks of age consisting predominantly of cDCs and macrophages, later followed (7–8 weeks) by a massive lymphocyte accumulation [8], [9] and a second wave of plasmacytoid DCs. At the time of this larger para- and peri-islet immune cell accumulation there is also a steady increase of dispersed cDCs and macrophages in the exocrine pancreas [8].

A key role for the peri-islet and pancreas accumulating cDC and macrophages in the pathogenesis of the destructive insulitis at 5 weeks of age is indicated by the demonstration that a temporal depletion of cDCs and macrophages at 5 weeks of age before the onset of lymphocytic insulitis blocks or significantly delays the diabetes onset in NOD mice [10], [11].

Two major cDC subsets with different phenotypes have been described in the lymphoid organs of mice: The tolerogenic CD8α^+^CD103^+^ cDCs that induce T cell apoptosis and the CD8α^−^CD11c^+^ cDCs that are strong inducers of CD4 T cell proliferation [12]. Both these subsets can also be found in the islets of the pancreas of mice [13], [14], where the CD8α^−^CD11c^+^cDCs form the majority of cDC, which accumulate from 5 weeks onwards around the islets of Langerhans [13], [15]. We have recently reported on reduced numbers of the minor population of tolerogenic CD8α^+^CD103^+^ DCs in the 5 week old pre-diabetic pancreas of NOD mice [16] and hypothesized that the reduced number of these tolerogenic DC contributes to the development of progressive destructive autoimmune insulitis.

In this report we focus on the larger subset of immunogenic CD11c^+^CD8α^−^ cDCs (from here referred to as CD8α^−^ DCs) in the pancreas of NOD mice of 5 weeks of age [13], [16]. We firstly analyzed this population versus a control pancreas CD8α^−^ DC population of the C57BL/6 mouse in a genome-wide gene expression analysis to elucidate abnormalities in gene expression networks. Abnormally expressed key genes in the networks were validated by Q-PCR and in functional assays such as cell proliferation assays and flow cytometric analysis (tolerance inducing genes).

To assess the responsiveness of the CD8α^−^ DCs to a danger signal, we stimulated NOD (and control C57BL/6) CD8α^−^ DCs isolated from the pancreas *in vitro* with LPS and measured the production of inflammatory cytokines; and in addition used whole genome analysis to measure changes in the networks of gene expression.

## Materials and Methods

### Mice

C57BL/6J and NOD/ShiLtJ female mice were purchased at Charles River Laboratories (Maastricht, The Netherlands). Mice were housed in groups (littermates) under specific pathogen-free conditions with a standard dark-light cycle and fed *ad libitum*. The diabetes incidence in female NOD mice is about 80%. All mice were euthanized by CO_2_ inhalation before collection of the tissues. All experimental procedures were approved by the Erasmus University Animal Welfare Committee in accordance with the Experiments on Animals Act (‘Wet op de dierproeven’).

### Preparation of cell suspensions

Pancreases of 5 week old mice were isolated after a cardiac perfusion, cut into small pieces and digested with Collagenase Type 1 (1 mg/ml), hyaluronidase (2 mg/ml) (both Sigma Aldrich, St. Louis, MO, USA) and DNAse I (0.3 mg/ml) (Roche Diagnostics, Almere, The Netherlands) for 40 minutes at 37°C. Subsequently, cells were flushed through a 70 µm filter and washed with DMEM +10% FCS. All cells were resuspended in PBS containing 0.1% BSA and were ready for flow cytometric staining.

Single-cell suspensions from pancreas were labeled with CD45 beads (Miltenyi, Leiden, The Netherlands) and CD45^+^ cells were pre-sorted with the AutoMACS pro (Miltenyi) to remove most of the non-immune cells. The pancreatic CD45^+^ cells were further processed for FACS analysis or DC isolation.

### Flow cytometry analysis

Subsequently, the CD45^+^ cells were labeled with mAbs on ice. Monoclonal antibodies for detection of pancreatic DCs were CD11c, CD24, CD8α, CD86 and CCR5 (all eBioscience, San Diego, CA, USA), CD11b-APC-Cy7 (Becton Dickinson, Breda, The Netherlands), CD200R3 (Hycult Biotech, Uden, The Netherlands). Afterwards cells were washed and fixed in PBS containing 0.1% BSA and 0.5% paraformaldehyde. Cell populations and marker expression were detected using a BD FACSCanto HTSII (Becton Dickinson) flow cytometer and analyzed with Flowjo software (Tree Star, Ashland, OR, USA).

### DC isolation and *in vitro* stimulation

The pre-sorted pancreatic CD45^+^ cells were labeled with CD11c and CD8α in PBS containing 0.1% BSA. Subsequently, CD8α^−^ DCs were sorted on a FACSAria II (Becton Dickinson). Figure S1 in File S1 shows the gating strategy. Re-evaluation of the sorted CD8α^−^ DCs indicated >98% purity of the sorted cells. Half of the total number of sorted CD8α^−^ DCs cells were washed and directly lysed in PicoPure extraction buffer (Arcturus, Applied Biosystems, Bleiswijk, The Netherlands) and stored at −80°C until RNA isolation procedure. The other half of CD8α^−^ DCs were cultured for 18 hours in RPMI-1640 medium supplemented with 10% FCS, 50 µM beta-mercaptoethanol and with or without 1 µg/ml LPS from *E. coli 0111:B4* (Sigma, Saint Louis, MO, USA). Finally cells were harvested with 2 mM EDTA and lysed in extraction buffer for RNA isolation. Supernatants were collected and stored at −80°C.

### RNA isolation, amplification and gene expression analysis

RNA was isolated with the PicoPure kit (Arcturus, Applied Biosystems) according to the manufacturer’s protocol including a DNase I treatment (Qiagen, Venlo, The Netherlands) to remove genomic DNA contamination. RNA quality was assessed on the bioanalyzer (Agilent Technologies, Amstelveen, The Netherlands) and samples with a RIN>8 were accepted. The RNA was reverse transcribed, amplified, biotinylated and fragmented with the Ovation Pico WTA v2 and Encore Biotin Module (NuGEN Technologies, Leek, The Netherlands) and subsequently hybridized on Mouse Genome 430 2.0 Arrays (Affymetrix, High Wycombe, UK) according to the manufacturers protocols. The raw data containing.CEL files, including metadata and matrix with normalized gene expression were uploaded to GEO and will be accessible from publication date under accession number: GSE45028 at http://www.ncbi.nlm.nih.gov/geo/query/acc.cgi?acc=GSE45028.

### Microarray analysis and qPCR validation

#### Microarray analysis

Quality analysis of the CEL data was assessed by running a standardized workflow developed at the BiGCaT department of Maastricht University - The Netherlands (http://www.arrayanalysis.org/). The expression data containing.CEL files were imported and processed further with BRB-ArrayTools (R. Simon, http://linus.nci.nih.gov/BRB-ArrayTools.html). Gene expression data was normalized using RMA (Robust Multichip Average) [17].

A list of differentially expressed genes (DEGs) among the two classes was identified by using a multivariate permutation test using the class comparison tool in BRB-arraytools. The multivariate permutation test was used to provide 90% confidence that the false discovery rate (FDR) was less than 10% [18], [19]. The FDR is the proportion of the list of genes claimed to be differentially expressed that are false positives. Partek Genomics Suite (Partek Inc., Saint Louis, MO, USA) was used for the principle component analysis (PCA) and for the hierarchically clustered representation of the DEGs. Ingenuity pathway analysis (Ingenuity Systems, www.ingenuity.com) was used for annotation, mapping of the DEGs to known biological networks and to visualize interactions between genes.

#### Quantitative PCR validation

RNA and cDNA for the Q-PCR validation was prepared according to the same procedure as described above. Q-PCR was performed with a commercially available mix (TaqMan Universal PCR Master Mix) according to the manufacturer’s protocol on a 7900HT Fast Real-Time PCR System (Applied Biosystems). All TaqMan probes and consensus primers were preformulated and designed by the manufacturer (TaqMan Gene Expression Assays; Applied Biosystems). The quantitative value obtained from Q-PCR is a cycle threshold (Ct). Normalized expression values for each gene were calculated by the following formula: 2^−(Ct[gene]–Ct[Rpl19])^, with *Rpl19* as housekeeping gene for normalization.

### BrdU incorporation and detection

Mice were injected intraperitoneal at an age of 5 weeks with 1 mg BrdU from the FITC BrdU flow kit (Becton Dickinson); Brdu (0.8 mg/ml) was added to the drinking water for the next 96 h hours. Mice were sacrificed after 24, 48 and 96 h and tissue was prepared described in the preparation of cell suspensions. The pre-sorted pancreatic CD45^+^ cells were stained with cell surface markers and subsequently fixed and permeabilized using Cytofix/Cytoperm and Perm/Wash buffer from the BrdU flow kit according to the manufacturer’s protocol. BrdU was detected by a monoclonal antibody BrdU-FITC (Becton Dickinson) BrdU expression in the pancreatic DC was detected using a BD FACSCanto HTSII (Becton Dickinson) flow cytometer and analyzed with Flowjo software (Tree Star).

### Cytokine measurements

Concentrations of IL-6, IL-10, IL-12p70 and TNF-α were measured in the supernatants from CD8α^−^ DC cultures with the FlowCytomix cytometric bead array according to the manufacturer’s protocol (eBioscience). Briefly, a mixture of beads coated with antibodies against IL-6, IL-10, IL-12p70 and TNF-α were incubated with the supernatant or standard mixture. The antigens present in the sample bind to the antibodies linked to the different fluorescent beads. A biotin-conjugated second antibody mixture was added and finally streptavidin-PE, to emit a fluorescent signal. Fluorescent signals from the beads were measured on a BD FACSCanto II HTS (Becton Dickinson) and analyzed with FlowCytomix Pro Software (eBioscience).

### Statistical analysis and figures

For direct comparisons between the strains, the Mann–Whitney U test was used for unpaired analysis or as noted otherwise in the figure legend. All analyses were carried out using IBM SPSS statistics 20 software (SPSS, Chicago, IL, USA) and considered statistically significant if P<0.05. Graphs were designed with Graphpad Prism 5.0 (Graphpad Software, La Jolla, CA, USA).

## Results

### Reduced expression of proliferation, maturation, migration, inflammation and growth factor gene networks in NOD steady state pancreatic CD8α^−^ DCs

To characterize the CD8α^−^ DC subset in the NOD, a microarray analysis was conducted on CD8α^−^ DCs isolated from 5 weeks old NOD and C57BL/6 pancreases. In total, 2122 differentially expressed genes (DEGs) among the pancreatic NOD and C57BL/6 CD8α^−^ DCs were identified, using a multivariate permutation test.

**Figure 1:**
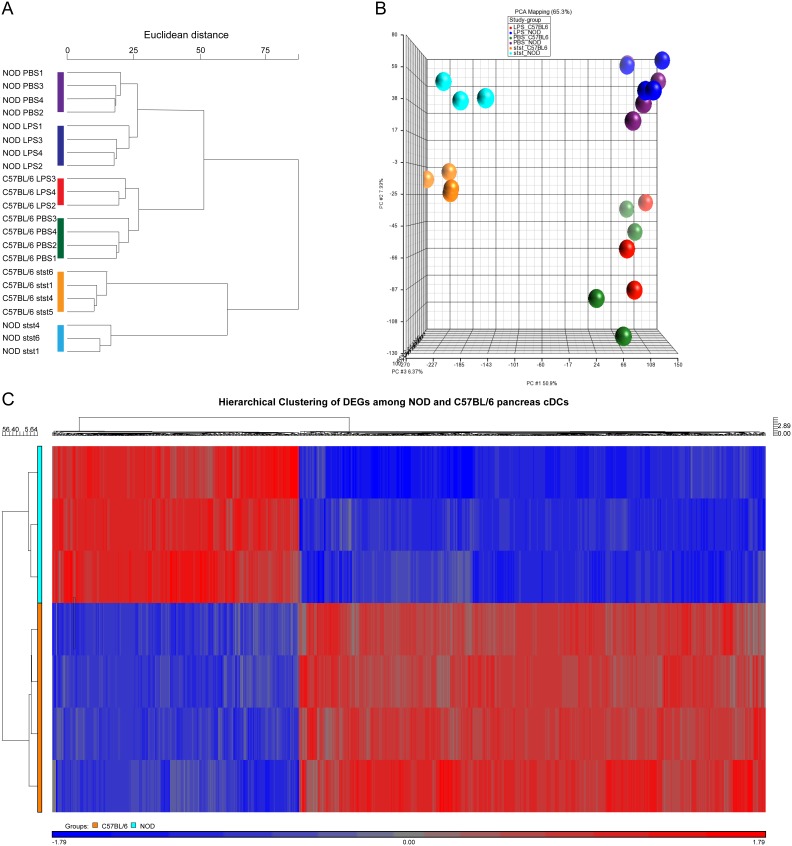
Graphical representation of the samples. Dendrogram of the samples after hierarchical clustering (Euclidean distance with average linkage) of the genome-wide gene expression profiles of the pancreatic CD8α^−^ DCs under steady-state conditions and after *in vitro* LPS stimulation (A). PCA analysis was used to compare all groups. Separation of the groups by principle components 1–3 that show 65.3% of the variance (B). Heatmap with hierarchical clustering (Euclidean distance with average linkage) of the DEG genes among the NOD (Cyan) and C57BL/6 (Orange) pancreatic CD8α^−^ DCs (C). Normalized 2 log-transformed probeset expression values are visualized as a gradient from low (blue) to high (red) expression.

Hierarchical clustering of the samples and PCA analysis indicated a clear distinction in global gene expression profiles between the NOD and C57BL/6 CD8α^−^ DCs (Figure 1a, b). The majority of DEGs (1380; 65%) were down-regulated in the NOD pancreas CD8α^−^ DCs. Figure 1c shows a hierarchically clustered heat map of all significant DEGs. We conducted Ingenuity Pathway analysis of the DEGs and mainly found a reduced expression of gene networks of proliferation/apoptosis factors, of maturation, migration and inflammatory response factors, and of growth factors including islet regeneration factors in NOD steady state pancreatic CD8α^−^ DCs. Table 1 shows the top abnormally expressed genes (lowest P-value, largest fold-up/down) in these networks as categorized by Ingenuity. Of the networks we validated several of these top-ranking genes in q-PCR (Table 1; in bold) and the q-PCR results confirmed the results obtained from the microarray analysis.

**Table 1:** Differentially expressed genes among the pancreatic C57BL/6 and NOD CD8α^−^ DCs.

Gene symbol	Fold change	P-value	Description
**Cell proliferation and apoptosis network**			
**Cdk1**	**−1.7**	**2.3e-4**	**cyclin-dependent kinase 1**
Cdk4			cyclin-dependent kinase 4
**Mki67**	**−2.4**	**1.0e-8**	**antigen identified by monoclonal antibody Ki-67**
Bcl2	**−**6.5	<1.0e-7	B cell leukemia/lymphoma 2
Bcl2a1a	**−**1.6	3.1e-4	B cell leukemia/lymphoma 2 related protein A1a
**Cell maturation, inflammation and cell migration network**			
Cd24	**−**1.4; −1.4; −1.5	8.4e-4; 4.4e-4; 4.7e-4	CD24a antigen
**Il10**	**−3.1**	**2.5e-5**	**interleukin 10**
Il12b	**−**2.8	1.6e-6	interleukin 12B
**Ifng**	**−2.5**	**5.5e-5**	**interferon gamma**
**Mx1**	**−1.9**	**8.6e-6**	**myxovirus (influenza virus) resistance 1**
IL15	**−**1.9	3.6e-4	interleukin 15
Il36g	1.9	7.0e-5	interleukin 1 family, member 9
**Ifi202b**	**35.0;14.1**	**<1.0e-7; <1.0e-7**	**interferon activated gene 202B**
**Ifi204**	**−26.0; −13.4; −12.9;** **−2.9; −2.7; −2.0**	**<1.0e-7; <1.0e-7;** **<1.0e-7; <1.0e-7;** **5.5e-6; 4.0e-7**	**interferon activated gene 204**
Ccl2	**−**2.1	2.3e-4	chemokine (C-C motif) ligand 2
**Ccr5**	**−4.9; −3.0; −1.9**	**2.0e-7; 2.3e-5; 9e-7**	**chemokine (C-C motif) receptor 5**
**Thbs1**	**−4.0; −2.9; −2.0**	**1.9e-4; 9.5e-5; 9.5e-5**	**thrombospondin 1**
**H60a**	**59.0; 5.6;**	**<1.0e-7; 1.0e-7**	**histocompatibility 60a**
**CD200R3**	**−34.2**	**<1.0e-7**	**CD200 receptor 3**
**Chi3l3 (or Ym1)**	**−1.9**	**6.9e-4**	**chitinase 3-like 3**
**CD40**	**−1.6; −1.5**	**1.5e-4;1.3e-4**	**CD40 antigen**
**Mif**	**1.3**	**2.7e-2**	**macrophage migration inhibitory factor**
**Growth and support networks**			
***Reg2***	**−34.9**	**<1.0e-7**	**Regenerating islet-derived 1 beta**
***Reg3a***	**−16.3; −4.2**	**<1.0e-7; 4.0e-7**	**Regenerating islet-derived 3 alpha**
***Reg3g***	**−3.9**	**1.0e-6**	**Regenerating islet-derived 3 gamma**
*Reg3d*	**−**2.4	1.8e-5	Regenerating islet-derived 3 delta
***Reg1***	**−2.4**	**7.6e-4**	**Regenerating islet-derived 1 alpha**
***Fgf2***	**−2.6**	**7.0e-7**	**Fibroblast growth factor 2**
**Others**			
Nrcam	41.2; 8.6, 5.1	<1.0e-7; <1.0e-7; <1.0e-7	Neuronal cell adhesion molecule
Cd209b	2.3; 2.0	1.9e-6; 6.3e-5	CD209b antigen ()
Ank2	3.9; 3.7; 3.7; 2.8; 2.5	<1.0e-7; <1.0e-7; 2.0e-7; 4.0e-7	ankyrin 2
Cacna1a	8.8; 6.2; 4.1; 2.8	<1.0e-7; <1.0e-7; 1.0e-7; 1.6e-4	calcium channel, voltage-dependent, P/Q type, alpha 1A subunit

Differentially expressed genes (DEGs) among the pancreatic C57BL/6 and NOD CD8α^−^ DCs were identified by using a multivariate permutation test. Negative fold change means down in NOD compared to C57BL/6 mice. The table shows a list of highly significant DEGs summarized per functional category. Annotation and functional category were provided by Ingenuity Pathway Analysis. Genes in **bold** were significant in the q-PCR validation.

### Proliferation/apoptosis network

**Figure 2:**
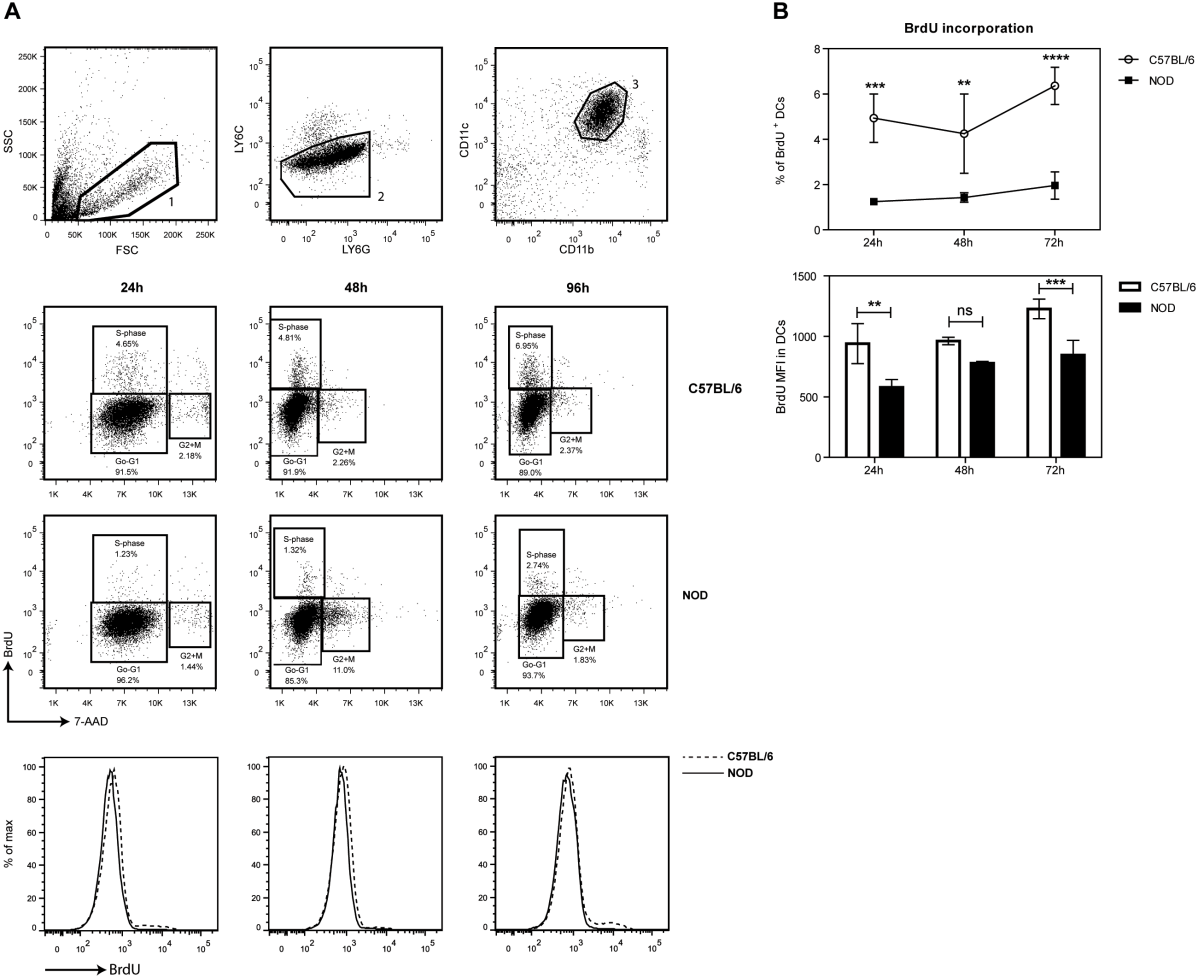
BrdU detection in mouse pancreas. Upper panel: gating strategy for DCs in the pancreas; bottom panel: FACS plots (top panel) containing the percentage of pancreatic DCs in the S-phase after 24, 48, 96 h in the 5 week old C57BL/6 and NOD mice. Histograms (bottom panel) showing the BrdU expression after 24, 48, 96 h in the C57BL/6 and NOD mice (A). Mean and SD of the percentages of BrdU^+^ DCs and mean and SD of the BrdU MFI in pancreatic DCs from both the NOD and C57BL/6 mice (B). N = 8, two-way ANOVA was used to compare % of BrdU+ cells over time and students t-test was used to compare BrdU MFI for each timepoint: **P<0.01, ***P<0.001, ****P<0.0001.

An important proportion of the discriminating cell proliferation and apoptosis DEGs were involved in down-regulation of proliferation, including a gene network involved in the proliferation of phagocytes (Figure S2 in File S1) suggesting a poor proliferation capacity of the DCs. To functionally verify a putative reduced proliferation capacity of NOD pancreatic DCs we injected NOD and C57BL/6 mice with BrdU, and BrdU^+^CD11c^+^ DCs in the pancreas were assessed by flow cytometric analysis after 24, 48, and 96 h (Figure 2a; CD8α was not included in the analysis as the vast majority of CD11c^+^ DCs are CD8α^−^). The total number of CD11c^+^BrdU^+^ DCs in the NOD pancreas was significantly decreased as compared to the C57BL/6 pancreas at all timepoints (Figure 2b), corroborating the finding of the reduced expression of genes involved in cell proliferation.

### Cell maturation, inflammation and cell migration network

A high ranking network within the reduced network of maturation and inflammation consisted of the down-regulation of various inflammatory response genes (Figure S3 in File S1), such as Il10, Il12b, Ifng and Chi3l3 (YM1, an enzyme involved in alternative macrophage polarization and known for its Th2 cell promoting effects [19], [20]) and the down-regulation of the co-stimulatory gene CD40. These findings suggest a reduced classical DC maturation of steady state NOD pancreatic CD8α^−^ DCs. This observation is supported by previous functional studies of our group, in which we found with regard to the DC differentiation from bone marrow precursors that the cells showed a poor differentiation into fully competent classical immune stimulatory DCs, yet deviated to another phenotype, i.e. a more “macrophage like” phenotype [20]. It is therefore important to note that we found in this network various inflammatory genes known for macrophage activation up-regulated (such as Ifi202b, Il36g and Mif) as well as CD209b. CD209b also known as SIGN-R1 is a C-type lectin involved in binding and capture of dextran, pathogens and encapsulated bacteria and highly expressed on macrophages [17]. Collectively these gene expression data point to a qualitatively different immune and inflammatory set point of the NOD CD8α^−^ DCs (more “macrophage-like”).

**Figure 3:**
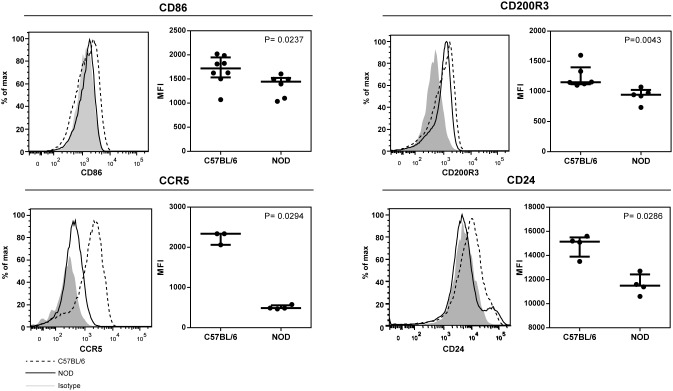
Flow cytometric analysis of CD8α^−^ DCs. Flow cytometric analysis of CD8α^−^ DCs isolated from the pancreas of 5 week old C57BL/6 and NOD mice according to the gating strategy shown in Figure 1. Histograms and scatterplots depict the expression of CD86, CD200R3, CCR5 and CD24 on CD11c^+^CD8α^−^ DCs. The median with error bars indicating the interquartile range are shown. (N = 4 for CCR5, N = 8 for the other markers). P-values were determined by Mann-Whitney U test.

We also found in the network of cell maturation, inflammation and cell migration the down-regulation of a set of genes involved in tolerance induction (CD200R3, CCR5 and CD24). To validate these findings we performed a limited flow cytometric analysis for the proteins encoded by these genes. NOD pancreatic CD8α^−^ DCs expressed minor, but significantly reduced levels of CD200R3, CCR5, and CD24 as well as for CD86 (Figure 3).

Several of the maturation and inflammatory network genes also belong to gene networks involved in migration of mononuclear leukocytes (e.g.Ccl2). The down-regulation of this network suggests a reduced migration/trafficking of the pancreatic CD8α^−^ DCs in the NOD mouse model. This is in line with the previous functional observations of our group on a reduced migration capability of NOD DC [21], [43].

### Growth and support networks

Of particular interest was also the down-regulation of growth-factor networks and a specific set of genes involved in islet regeneration belonging to the Islet Regenerating (Reg) gene family, which were strongly down-regulated in the NOD pancreatic CD8α^−^ DCs (Table 1). These genes included Reg1, 2, 3a, 3d and 3g.

On the other hand various genes important in interaction with neurons were found upregulated and of these neuronal cell adhesion molecule (NRCAM) was highly significant. NRCAMs play a role in neuronal cell adhesion and axon guidance, but is also expressed in the pancreas [18]. Other molecules that have been described as important in neuron interaction and that were found up-regulated were neuronal Ank2 and Cacna1 (Table 1). Interactions of DCs and macrophages with islet nerves in the early phases of the NOD insulitis are well documented [21], [22].

### NOD pancreas-derived steady state CD8α^−^ DCs are hyperreactive to LPS stimulation with regard to the up-regulation of inflammatory response genes

We continued to analyze by microarray analysis the responsiveness of the pancreas CD8α^−^ DC subset of both the NOD and C57BL/6 mouse to *in vitro* inflammatory stimulation with LPS for 18 hours (with PBS as control). Hierarchical clustering of the samples resulted in two clusters representing the mouse strain, each containing two sub-clusters representing LPS stimulation (Figure 1a). In addition, the PCA showed 6 clusters: two separate clusters indicating the DCs under steady state conditions (cyan and orange spheres), and four clusters representing the *in vitro* PBS/LPS-stimulation of the DCs for both mouse strains (Figure 1b).

**Figure 4:**
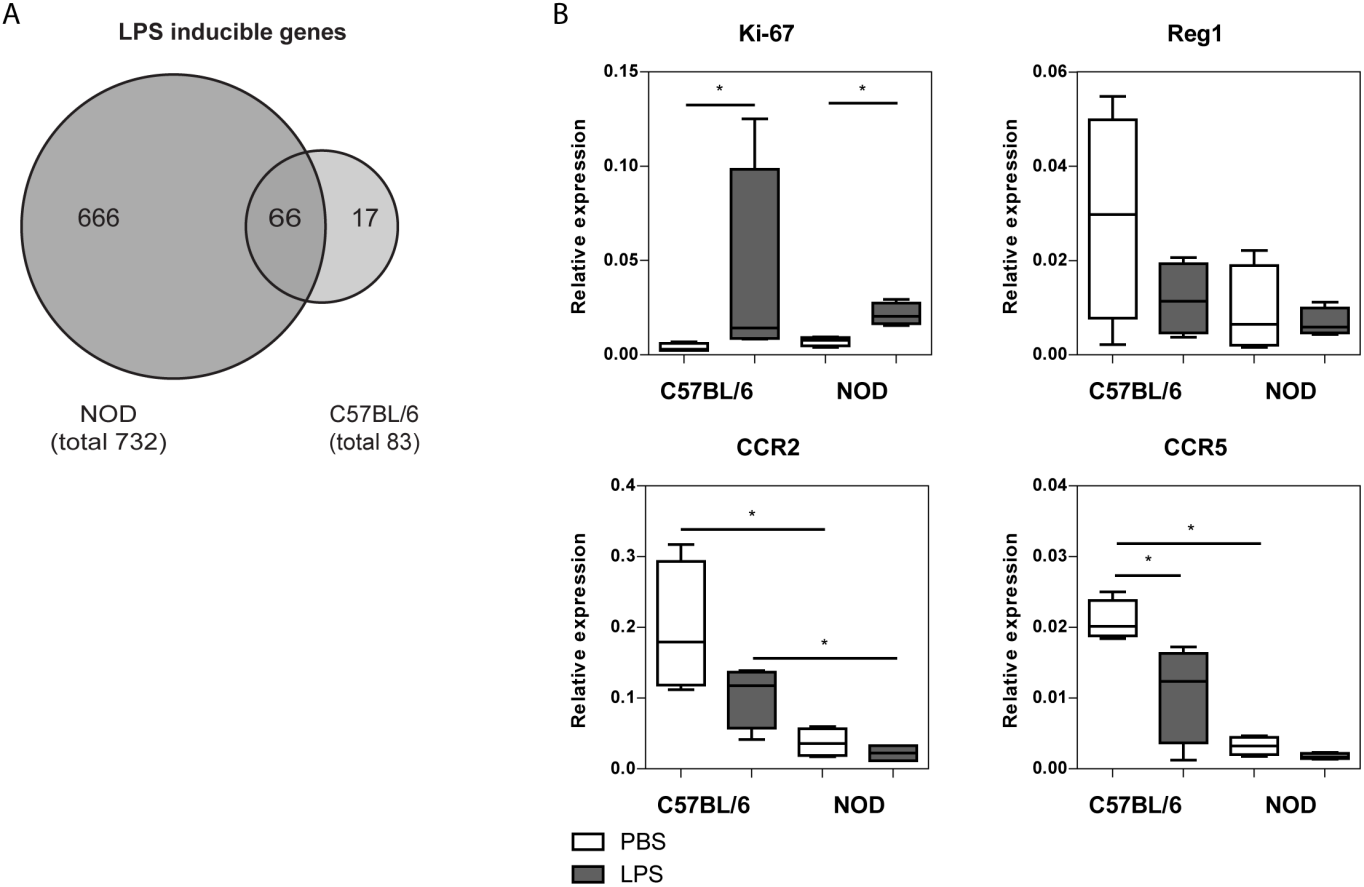
LPS inducible genes in NOD and C57BL/6 pancreatic DCs. DCs were *in vitro* stimulated for 18 h with LPS (or PBS). LPS-inducible genes for each strain were identified were identified by using a multivariate permutation test. The venn diagram shows the total number of LPS-inducible genes for each strain, the common LPS-inducible genes and the unique LPS-inducible genes per strain (A). DCs were isolated from C57BL/6 and NOD pancreas at an age of 5 weeks. DCs were cultured for 18 h in the presence of LPS or under control conditions (PBS). Relative expression of Ki67, Reg1, CCR2 and CCR5 were measured by Q-PCR (B). The box represents the interquartile distance, with a line on the median. The wiskers represent the 5 to 95 percentiles; N = 6 of 10 pooled mice per sample, P-values were determined by Mann-Whitney U test; *p<0.05.

Multivariate permutation testing was used to identify DEGs among the PBS- and LPS-stimulated CD8α^−^ DCs obtained from either NOD or C57BL/6 pancreases. A total number of 66 common LPS-responsive genes were identified in the NOD and C57BL/6 pancreatic CD8α^−^ DCs (Figure 4a). Ingenuity pathway analysis indicated that these genes were mainly involved in inflammatory responsiveness with a strong up-regulation of genes such as Il10, Il1b and Ptgs2 (Table 2 and Figure S4 in File S1). In addition, a unique pattern of LPS-responsive genes was identified for each mouse strain (Figure 4b). A larger number of LPS-responsive genes was identified in the NOD (666 in total) pancreas CD8α^−^ DCs compared to C57BL/6 (17 in total), suggesting that NOD DCs are more sensitive to the effect of LPS. This was confirmed by the PCA analysis (Figure 1b); there is a clear distinction between the *in vitro* PBS/LPS-treated NOD DCs (blue and purple spheres) in contrast to the C57BL/6 DCs (red and green spheres). There were no differences in the expression of toll-like receptor 4 on both the C57BL/6 as well as the NOD CD8α^−^ DCs (*data now shown*). Ingenuity pathway analysis indicated that indeed the LPS-inducible genes unique for the NOD pancreatic CD8α^−^ DCs were involved in inflammatory responsiveness including TREM-1 signaling. Of particular interest were the cytokines: Il6, Csf2 and Tnf, which were all specifically up-regulated in the *in vitro* LPS-stimulated NOD pancreatic CD8α^−^ DCs (Table 2).

**Table 2:** Differentially expressed genes among the *in vitro* LPS-stimulated pancreatic C57BL/6 and NOD CD8α^−^ DCs.

Gene symbol	Fold change	P-value	Description
**Common LPS-inducible genes**			
***Il1b***	**3.3**	**3.3e-6**	**interleukin 1 beta**
***Ptgs2***	**1.7**	**9.2e-4**	**prostaglandin-endoperoxide synthase 2**
***Il10***	**4.1**	**2.0e-7**	**interleukin 10**
*Il1f6*	6.5	1.4e-5	interleukin 1 family, member 6
*Il12a*	2.9	1.5e-6	interleukin 12a
***Mki67***	**2.5**	**1.9e-5**	**antigen identified by monoclonal antibody Ki-67**
**NOD specific LPS-inducible genes**			
**Il6**	**3.0**	**1.4e-5**	**interleukin 6**
**Tnf**	**1.7**	**5.7e-5**	**tumor necrosis factor**
**Csf2**	**9.8**	**3.0e-6**	**colony stimulating factor 2 (granulocyte-macrophage)**
Trem1	2.1	4.5e-4	triggering receptor expressed on myeloid cells 1
Trem2	**−**2.2	2.4e-5	triggering receptor expressed on myeloid cells 2
Itgax (CD11c)	**−**1.5	1.6e-3	integrin, alpha X (complement component 3 receptor 4 subunit)
Stat5a	1.8	2.0e-4	signal transducer and activator of transcription 5A
Tlr4	**−**1.6	3.5e-4	toll-like receptor 4

Differentially expressed genes (DEGs) among the *in vitro* LPS-stimulated pancreatic C57BL/6 and NOD CD8α^−^ DCs were identified by using a multivariate permutation test. The table shows a list of highly significant DEGs summarized per functional category. Annotation and functional category were provided by Ingenuity Pathway Analysis. Genes in **bold** were also significant in the q-PCR validation.

**Figure 5:**
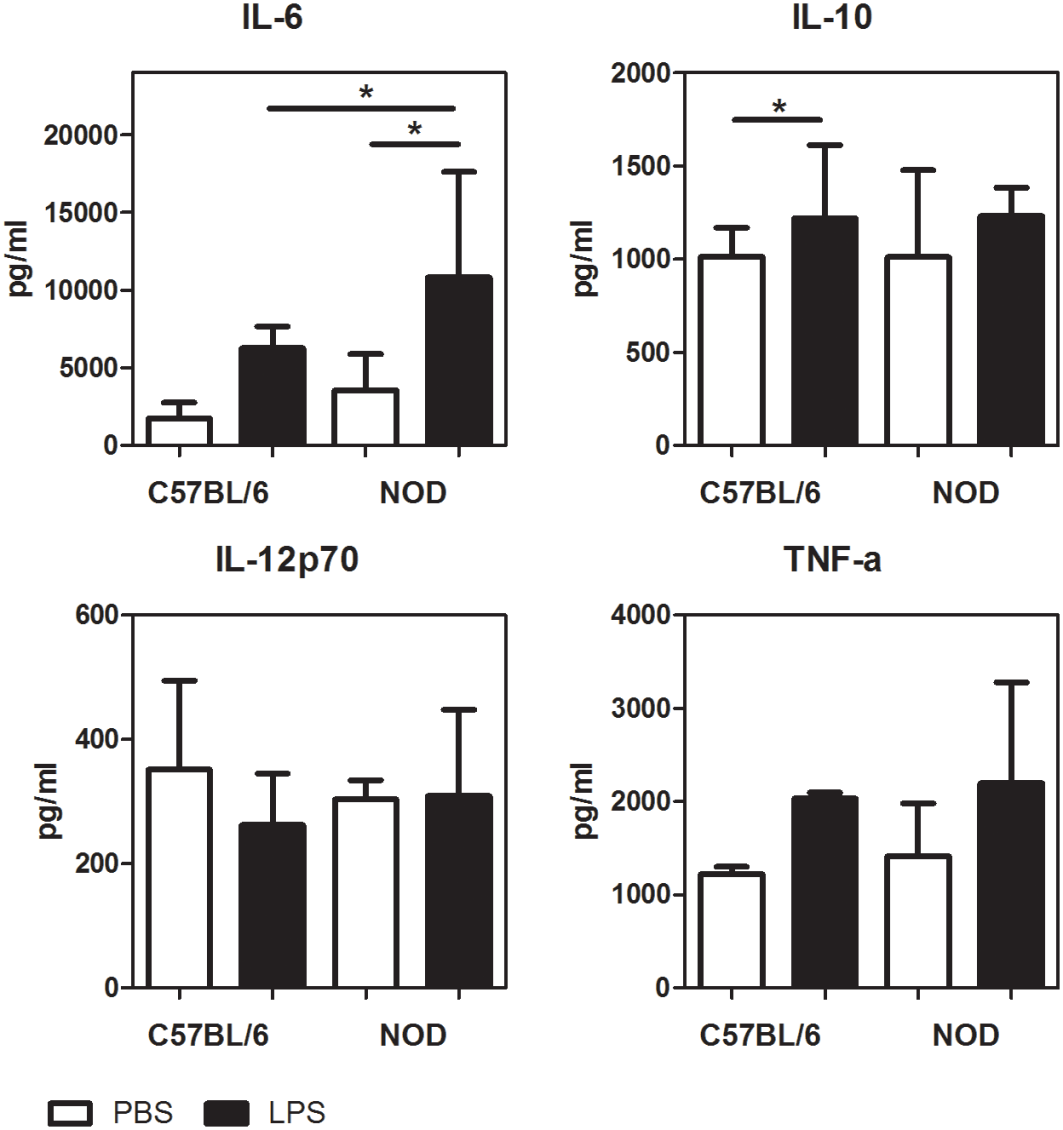
Cytokine production by *in vitro* LPS-stimulated pancreas DCs. DCs were isolated from C57BL/6 and NOD pancreas at an age of 5 weeks. DCs were cultured for 18 h in the presence of LPS or under control conditions (PBS). IL-6, IL-10, IL-12p70 and TNF-α were measured in the supernatant. Bars are represented as median with IQR with N = 4 of 10 pooled mice per sample, P-values were determined by Mann-Whitney U test; *p<0.05.

We therefore additionally measured a panel of cytokines in the supernatant of the LPS-stimulated pancreatic CD8α^−^ DCs. LPS did not stimulate the production of IL-12 (Figure 5). IL-10 and TNF-α production were increased after LPS stimulation (as in gene expression), but only reached statistical significance for IL-10 in the CD8α^−^ DCs from the C57BL/6 pancreas. IL-6 production was stimulated by LPS and there was a small, but significant increase in IL-6 concentration in LPS stimulated NOD pancreatic CD8α^−^ DCs as compared to PBS stimulated DCs and as compared to the LPS-stimulated CD8α^−^ DCs isolated from the C57BL/6 pancreas (Figure 5). These cytokine production findings of a slightly higher IL-6 production support the view that NOD CD8α^−^ DCs are hyperreactive to LPS stimulation, though do not show a highly excessive pro-inflammatory cytokine production from LPS stimulated NOD CD8α- DCs.

With regard to the other gene networks found repressed in NOD steady state pancreas CD8α^−^ DCs, such as the proliferation network and the network of growth factor genes for islets, these networks were neither hyperreactive to LPS, nor different anymore between NOD and C57BL/6 under LPS conditions. It must be noted that in general LPS stimulation reduced the production of REG gene expression (although not significant) (Figure 4b). Genes important in the migration network, such as CCR2 and CCR5, stayed repressed in the NOD CD8α^−^ DCs in comparison to C57BL/6 CD8α^−^ DCs (Figure 4b) after LPS stimulation.

## Discussion

Previously, we showed in a number of studies [20], [23] that DC generation from NOD bone marrow precursors resulted in a low yield of DCs that had various macrophage characteristics, such as a high acid phosphatase content. These DCs were defective in stimulating T cells. On the other hand – and in contradiction - there are also a number of reports of other investigators showing that DCs generated from NOD bone marrow precursors have elevated co-stimulatory, IL-12 and NF-κB activation resulting in an enhanced stimulatory function and in Th1 skewing abilities [24]–[26].

Also in type 1 diabetic (T1DM) patients discrepancies with regard to the differentiation and maturation state of DCs have been reported [27]. We described in 1995 a defective maturation and stimulatory function of DCs derived from monocytes in T1DM patients [28], an observation which was supported by later studies of Takahashi *et al* and Skarsvik *et al* who also found the defects in pre-diabetic individuals [29], [30]. However Zacher *et al.* did not find gross differences between monocyte-derived DCs of T1DM patients and healthy controls, with the limited discrepancies observed actually suggesting an enhanced maturation of the cells in T1DM [31]. Peng *et al.* also found signs of an activation of DCs in T1DM and described higher numbers of more mature circulating DCs as determined by flow cytometric analysis of the peripheral blood of recent onset T1DM patients [32]. However Vuckovic *et al.* using a similar methodology found decreased dendritic cell counts in children with recent onset T1DM [33].

This report on NOD pancreatic CD11c^+^CD8α^−^ DCs provides greater insight into the above described discrepancies. It shows that the major subset of steady state DCs isolated from the early pre-diabetic NOD pancreas, the CD8α^−^ DCs, has an altered gene expression set point and a reduced expression of several molecular networks important for the prime functions of the cell, such as cell renewal, immune tolerance induction, migration and the provision of growth factors for beta cell regeneration. This generally reduced expression state was easily switched over to hyper stimulation: The NOD steady state CD8α^−^ DCs were hyperreactive to the danger signal LPS resulting in a state of gene expression with a number of classical pro-inflammatory factors and cytokine genes excessively raised which were particularly down in steady state. We also found some indications that this pro-inflammatory hyperreactivity occurred at the protein level: In the limited set of cytokine production experiments carried out, IL-6 production profiles from NOD LPS stimulated CD8α^−^ DCs supported a hyperreactivity towards LPS. Interestingly a hyperproduction of inflammatory cytokines of NOD macrophages upon encounter of another danger-associated molecular patterns (DAMP), i.e. upon encounter with apoptotic or necrotic cells, has been described before [34]. It is tempting to speculate that the reduced expression of CD24 on the defective CD8α^−^ DCs found here plays a key role in the exaggerated switch of the DCs to the pro-inflammatory state. CD24 represses DAMP-signal-induced immune responses and CD24 deficient mice display massive increases in pro-inflammatory cytokines [35].

Our study is in fact the first study to assess gene expression profiles of CD8α^−^ DCs isolated from the pre-diabetic steady-state pancreas. Kodama *et al*. (2008) and Wu *et al*. (2012) studied gene expression profiles in splenocytes isolated from pre-diabetic NOD mice, (a mixture of leukocytes, mainly including lymphocytes, macrophages and DCs). These authors also found various abnormal gene expression patterns partly overlapping with ours, particularly the proliferation and immune response gene expression profiles [36], [37]. Also, Kodama *et al.* and Wu *et al*. found the majority of genes to be repressed as compared to normal mice. The authors observed that a large part of the abnormally expressed genes were coded for in the diabetes susceptibility regions, the *Idd* chromosomal loci, and they suggested that this might explain the abnormal expression. Indeed, some of our highly significant abnormally expressed genes (the MHC II-class related genes and the Fgf2 gene) are also part of these loci. In contrast, Wu *et al.* found several abnormalities in metabolic and enzymatic activity pathways, which we did not observe in the present study. It is likely that the heterogeneity of cell types in the spleen might play a significant role in this discrepant outcome, because less than 10% of total splenocytes are DC.

Our present and previous data [38] and also those of others [39] strongly suggest a deficiency in the proliferation, differentiation and maturation capabilities of steady state NOD mouse DCs both systemically and in the pancreas. This general “immune deficiency-like state” probably not only affects the effector immune functions of the DCs, but also their tolerance inducing capabilities, since we found important molecules playing a role in tolerance induction (CD24, CD200R3) to be down-regulated on the steady state pancreas CD8α^−^ DCs of the NOD mouse. Previous experiments with adoptive transfers of mature bone-marrow derived DCs expressing high levels of co-stimulatory molecules, such as CD80, CD86 and CD40, significantly reduced diabetes incidence in NOD mice [40]. Interestingly, treatment with immature DCs that express low levels of co-stimulatory molecules did not protect against diabetes [41] indicating that overcoming the poor differentiation and maturation state of DCs is of key importance for protection from diabetes in NOD mice.

Novel is our finding of the down-regulation in NOD pancreas CD8α^–^ DCs of a network of important genes for beta cell regenerating growth factors, the REG genes. REG genes were initially discovered for their role in the generation of beta cells in the human, rat and mouse pancreas [42]–[44]. DCs and macrophages play an important role in islet development [6] and it is tempting to speculate that the REG produced by these cells is instrumental in this support function. The down-regulation of REG genes in the deficient NOD pancreas DCs might in such view result in an insufficient support for islet growth and the aberrant islet morphogenesis that has been observed in the NOD pancreas from birth onwards prior to the first signs of lymphocytic insulitis [6], [45]. Whether an insufficient provision of REG growth factors also plays a role in the actual disappearance of the beta cells in the insulitis phase is not known. There is an increased expression of Reg2 in the total pancreas of NOD mice during diabetes development and staining of the 10-week old NOD pancreas showed expression of Reg 1, 2 and Reg3α, -γ proteins in the islets. In addition, all REG genes seem to have an IL-6 responsive element and treatment of healthy human islets with IL-6 results in increased REG production [46]. It is therefore not surprising that adjuvant immunotherapy increased expression of Reg2 which resulted in regeneration of beta cells [47].

Although this study has several limitations: e.g. the pancreas enzymatic digestion method and the low yield of cells making only limited (flow cytometric) and *in vitro* cytokine production studies possible. Another limitation is the CD11c^+^CD8α^−^ cell population might contain a small fraction plasmacytoid DCs. We are still confident that we can conclude that the gene expression profiles together with the limited flow cytometric data and our previous functional data support a view that under steady state conditions the CD8α^−^ DCs in the 5 week old pancreas of the NOD mouse display an altered phenotype with reduced cell renewal, migration, maturation and tolerance induction capabilities. These altered steady state NOD DCs are hyperresponsive to a danger stimulus, leading to a DC type with an exaggerated inflammatory molecular profile.

## Supporting Information

File S1 Supporting file containing: **Figure S1.** CD11c^+^CD8α^−^ DCs subset in the pancreas of C57BL/6 and NOD mice. **Figure S2.** Down regulation of phagocyte proliferation network under steady-state conditions. **Figure S3.** Down regulation of inflammatory response network under steady-state conditions. **Figure S4.** Inflammatory response network after in-vitro LPS stimulation.
(DOCX)
