# Supplementary material for: The Gene Expression Profile of CD11c+CD8α− Dendritic Cells in the Pre-Diabetic Pancreas of the NOD Mouse
Source: PLoS One. 2014 Aug 28;9(8):e103404. doi: 10.1371/journal.pone.0103404 (PMC4148310; doi:10.1371/journal.pone.0103404)
Supplement: File S1 — Supporting file containing: Figure S1. CD11c+CD8α− DCs subset in the pancreas of C57BL/6 and NOD mice. Figure S2. Down regulation of phagocyte proliferation network under steady-state conditions. Figure S3. Down regulation of inflammatory response network under steady-state conditions. Figure S4. Inflammatory response network after in-vitro LPS stimulation. (DOCX) [file pone.0103404.s001.docx]

## Figure S1


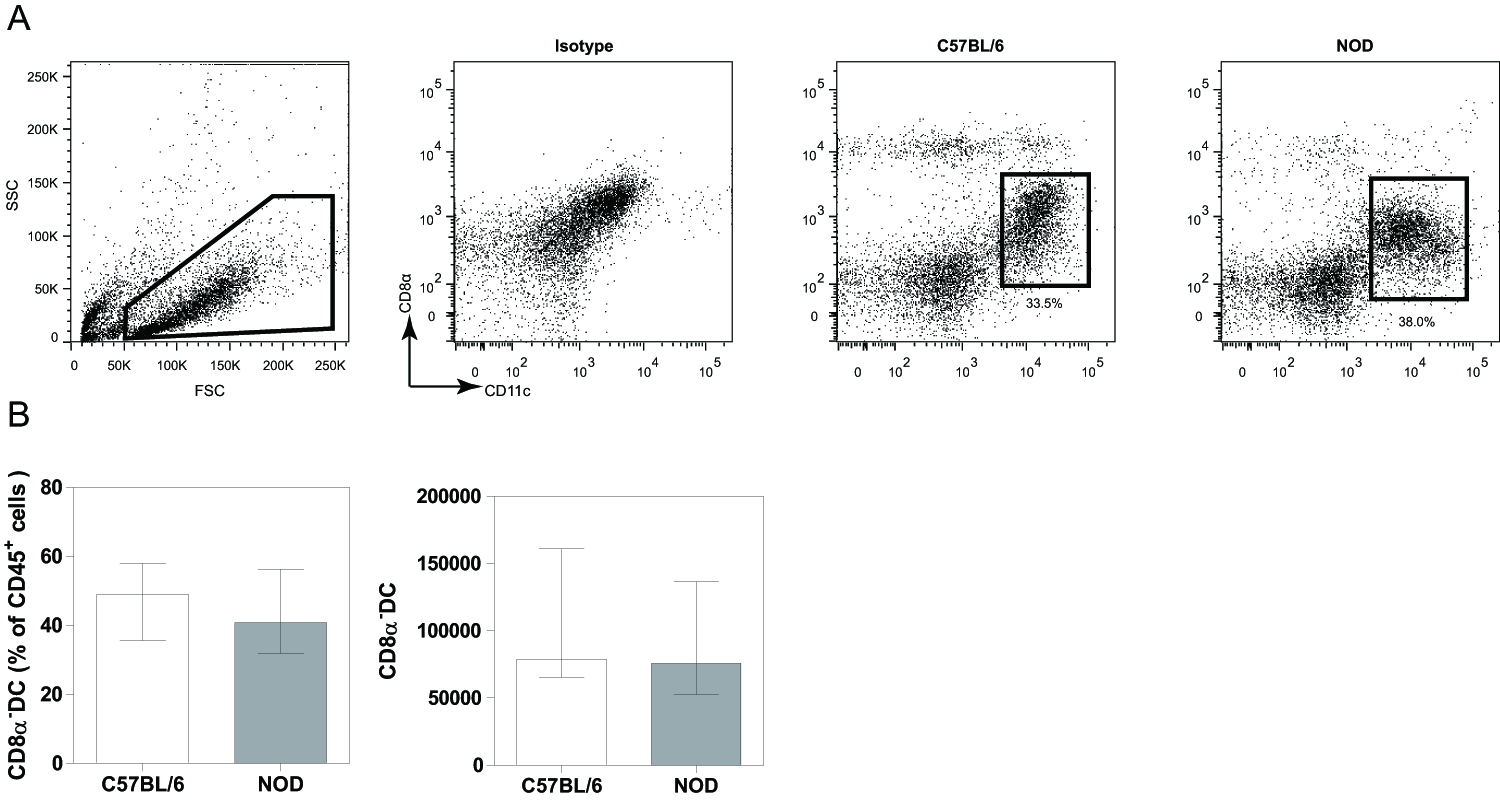


**Figure S1: CD11c^+^CD8α^-^ DCs subset in the pancreas of C57BL/6 and NOD mice.** Gating strategy of the pancreatic DCs (A). Percentage of CD11c^+^CD8a^-^ DCs of the total number of CD45^+^ cells and absolute number of CD11c^+^CD8a^-^ DCs in the pancreas of 5 week old C57BL/6 and NOD mice (B). There was no significant difference in percentage or absolute number of CD11c^+^CD8a^-^ DCs. (n=8); Bars represent the median value with IQR; P-values were determined by Mann-Whitney U test.

## Figure S2


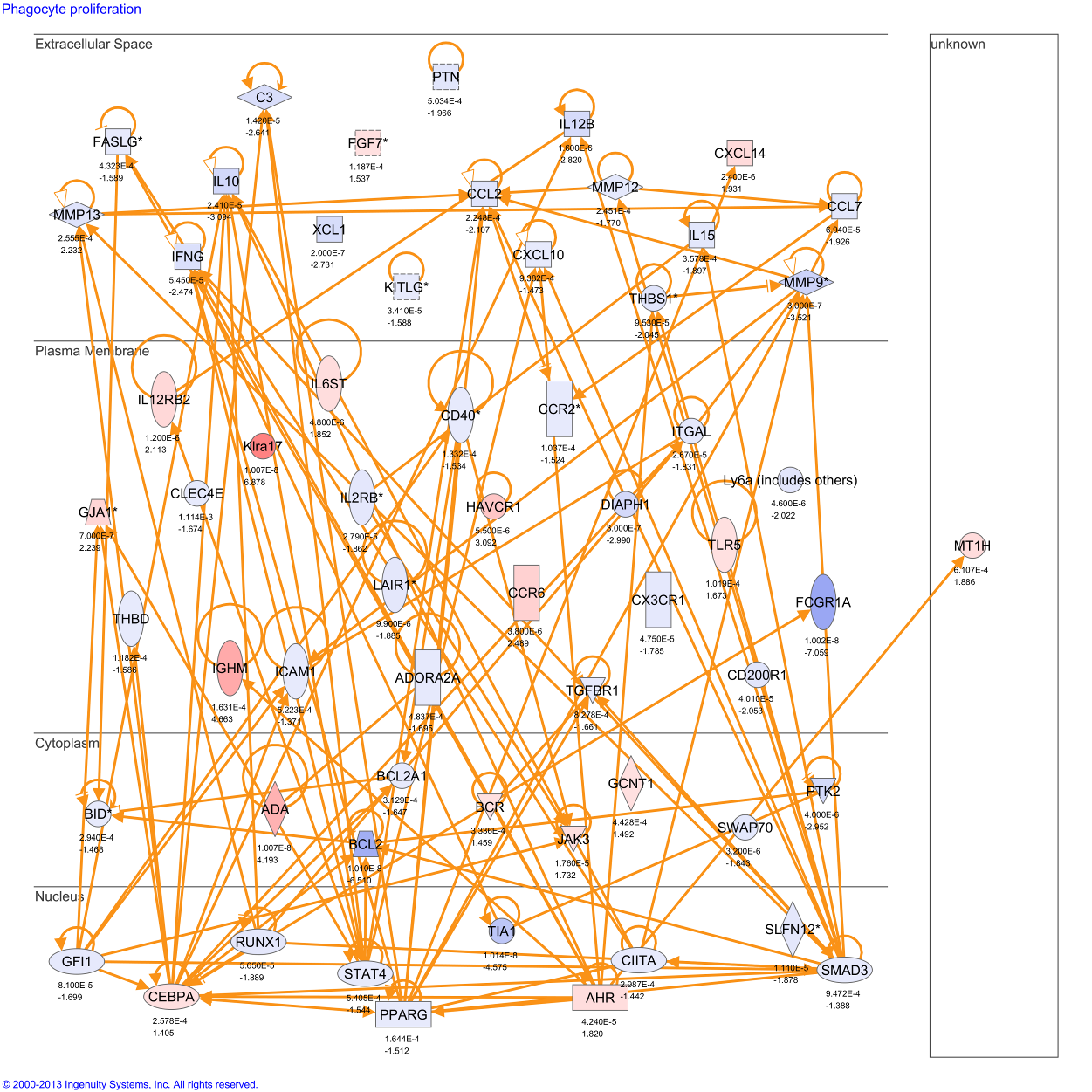


**Figure S2: Down regulation of phagocyte proliferation network under steady-state conditions.** Subcellular layout of phagocyte proliferation network identified by Ingenuity Pathway Analysis of DEG among the pancreatic C57BL/6 and NOD CD8α^-^ DCs. The z-score of this network is -1.2 with a p-value of 7.2e-5 indicating a significant down regulation of phagocyte proliferation. Genes that were up regulated are depicted in red and down regulated genes in blue. P-value and fold change for each specific gene are shown below each gene. Orange arrows depict experimentally observed and published (peer-reviewed) relationships between the genes.

## Figure S3


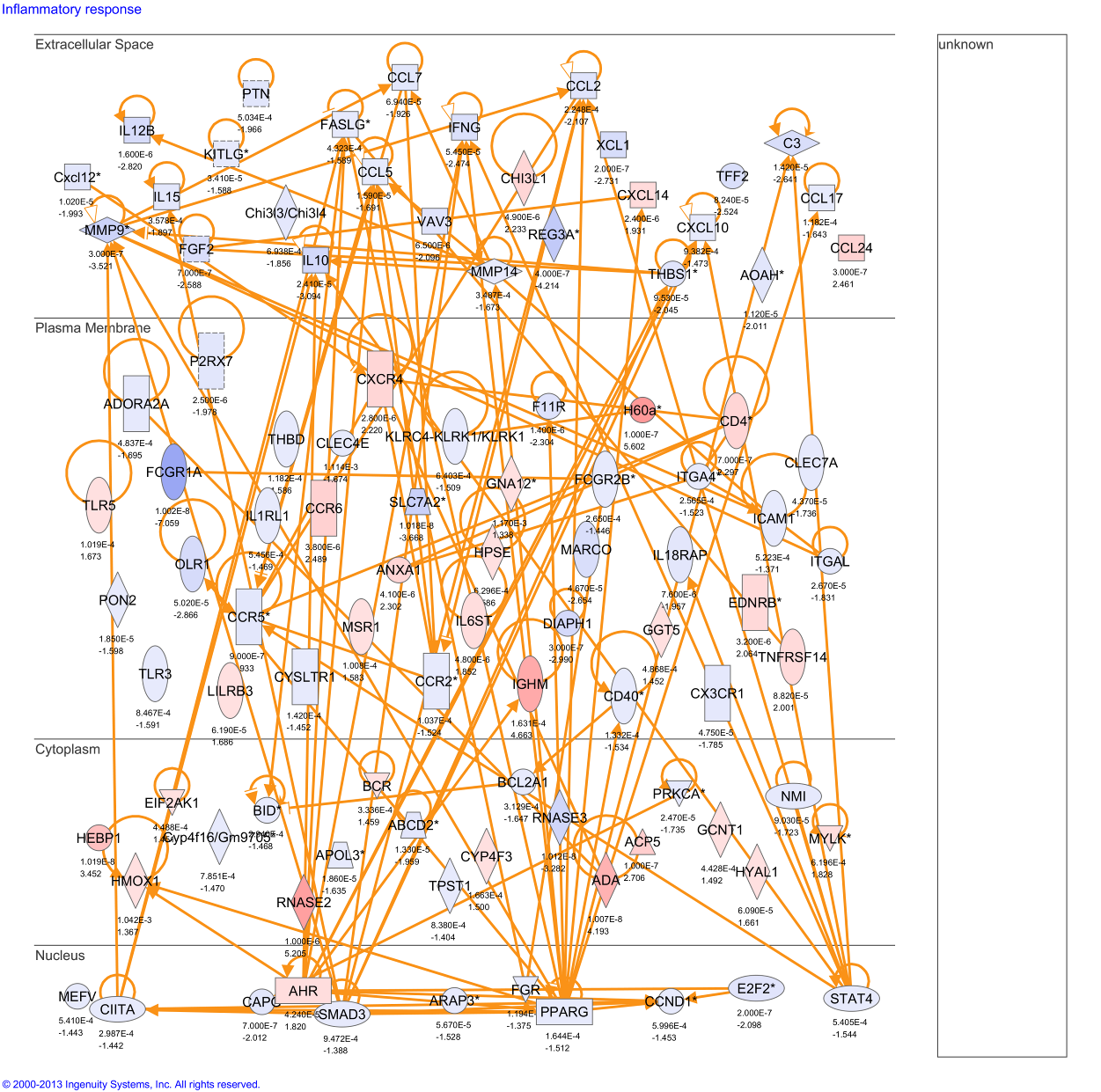


**Figure S3: Down regulation of inflammatory response network under steady-state conditions.** Subcellular layout of inflammatory response network identified by Ingenuity Pathway Analysis of DEG among the pancreatic C57BL/6 and NOD CD8α^-^ DCs. The z-score of this network is -2.6 with a p-value of 1.5e-5 indicating a significant down regulation of the inflammatory response. Genes that were up regulated are depicted in red and down regulated genes in blue. P-value and fold change for each specific gene are shown below each gene. Orange arrows depict experimentally observed and published (peer-reviewed) relationships between the genes.

## Figure S4


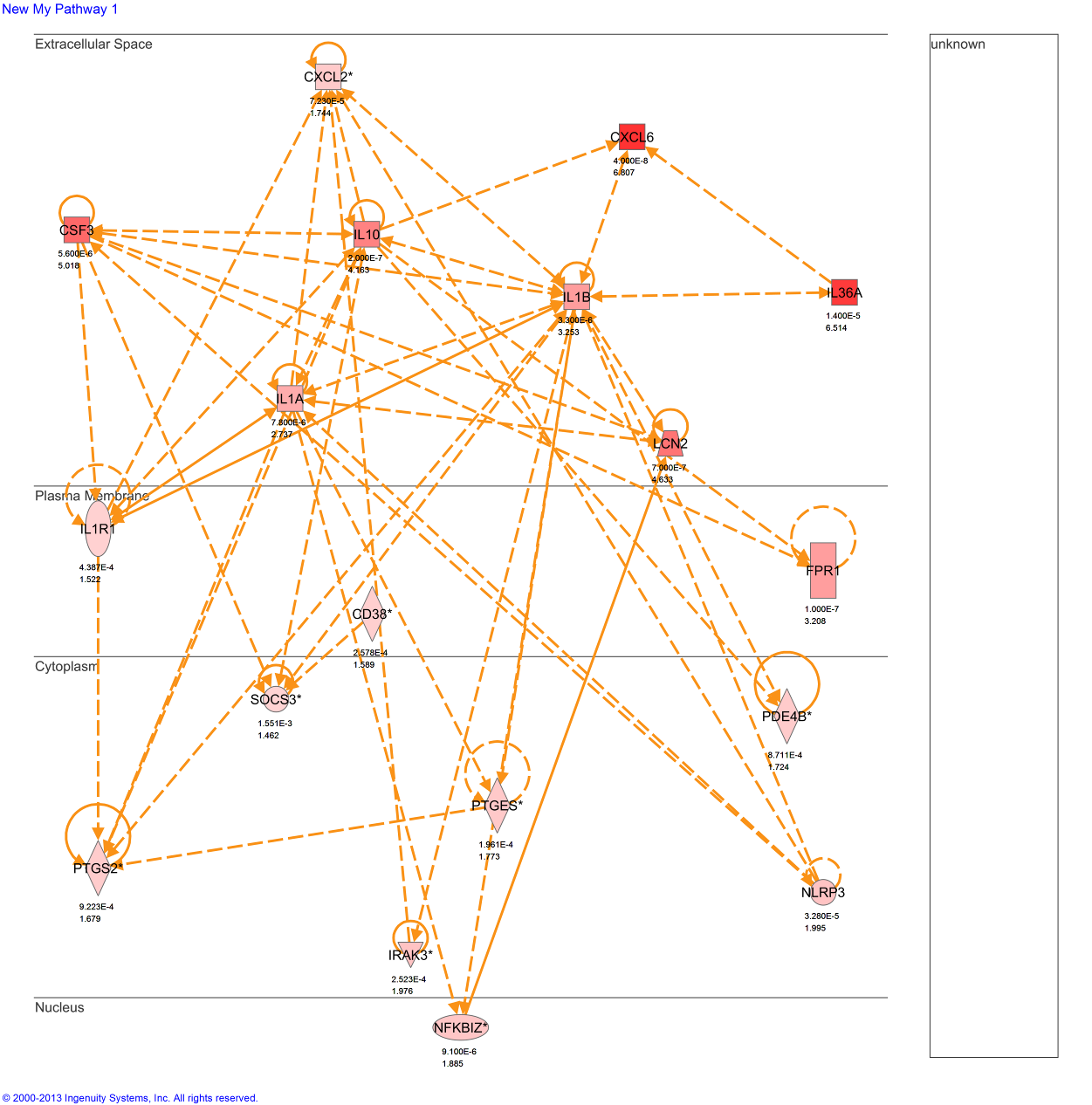


**Figure S4: Inflammatory response network after *in-vitro* LPS stimulation.** Subcellular layout of inflammatory response network identified by Ingenuity Pathway Analysis of common LPS-inducible genes. Genes that were up regulated are depicted in red. P-value and fold change for each specific gene are shown below each gene. Orange arrows depict experimentally observed and published (peer-reviewed) relationships between the genes.
